# Attribution of invasive group A streptococcal infections (iGAS) to predisposing viral infections, the Netherlands, 2010 to 2023

**DOI:** 10.2807/1560-7917.ES.2024.29.40.2300739

**Published:** 2024-10-03

**Authors:** Brechje de Gier, Jan van de Kassteele, Liselotte van Asten, Annelot F Schoffelen, ISIS-AR study group, Mariette Hooiveld, Margreet JM te Wierik, Nina M van Sorge, Hester E de Melker, Members of the ISIS-AR study group, JWT Cohen Stuart, DC Melles, K van Dijk, A Alzubaidy, M Scholing, SD Kuil, GJ Blaauw, W Altorf van der Kuil, SM Bierman, SC de Greeff, SR Groenendijk, R Hertroys, JCM Monen, DW Notermans, J Polman, WJ van den Reek, C Schneeberger-van der Linden, F Velthuis, CCH Wielders, BJ de Wit, RE Zoetigheid, W van den Bijllaardt, EM Kraan, MB Haeseker, JM da Silva, E de Jong, B Maraha, AJ van Griethuysen, BB Wintermans, MJCA van Trijp, AE Muller, M Wong, A Ott, E Bathoorn, M Lokate, J Sinnige, DC Melles, N Plantinga, NH Renders, JW Dorigo-Zetsma, LJ Bakker, K Waar, MT van der Beek, MA Leversteijn-van Hall, SP van Mens, E Schaftenaar, MH Nabuurs-Franssen, I Maat, PDJ Sturm, BMW Diederen, LGM Bode, DSY Ong, M van Rijn, S Dinant, M den Reijer, DW van Dam, EIGB de Brauwer, RG Bentvelsen, AGM Buiting, ALM Vlek, M de Graaf, A Troelstra, AR Jansz, MPA van Meer, J de Vries, JD Machiels

**Affiliations:** 1 Center for Infectious Disease Control, National Institute for Public Health and the Environment (RIVM), Bilthoven, the Netherlands; 2 The members of the ISIS-AR study group are listed under Collaborators.; 3 Nivel, Utrecht, the Netherlands; 4 Netherlands Reference Laboratory for Bacterial Meningitis, Amsterdam University Medical Center location AMC, Amsterdam, the Netherlands; 5 Department of Medical Microbiology and Infection Prevention, Amsterdam University Medical Center location AMC, University of Amsterdam, Amsterdam Institute for Infection and Immunity, Amsterdam, the Netherlands

**Keywords:** Streptococcus pyogenes, Influenza, Varicella, timeseries analysis

## Abstract

**Background:**

After most COVID-19 pandemic control measures were lifted in 2022, many infectious diseases re-emerged. An increase in invasive group A streptococcal (iGAS) infections among adults and young children was reported by several countries. Viral infections including influenza and varicella, known risk factors for iGAS infection, also increased.

**Aim:**

To estimate the proportion of GAS skin and soft tissue infections (SSTI) and pneumonia/sepsis in children (≤ 5 years) attributable to varicella, and the proportion of GAS pneumonia/sepsis in children and adults attributable to potentially predisposing respiratory viruses influenza A and B, RSV, hMPV and SARS-CoV-2 in the Netherlands.

**Methods:**

We performed time series regression using weekly data on respiratory viruses, varicella and non-invasive GAS infections and GAS isolates cultured from blood, lower airways, skin, pus and wounds, from January 2010 to March 2023.

**Results:**

In 2010–19, 50% (95% CI: 36–64) of GAS SSTI in children were attributable to varicella. Between January 2022 and March 2023, 34% (95% CI: 24–43) of GAS SSTI cases were attributable to varicella. Of iGAS pneumonia/sepsis between January 2022 and March 2023, 34% (95% CI: 20–49) and 25% (95% CI: 18–32) was attributable to respiratory virus infections in children and adults, respectively, with the largest contributor (17%) being influenza A.

**Conclusions:**

Predisposing viral infections likely contributed to, but cannot fully explain, the observed iGAS increase among children and adults in 2022–23 in the Netherlands. Public health measures to control viral infections, such as vaccination against varicella or influenza, might reduce the iGAS disease burden.

**Key public health message**

**What did you want to address in this study and why?**

Invasive group A streptococcal infection (iGAS) is a rare but severe disease, which can cause sepsis, pneumonia and skin or soft tissue infections. After the COVID-19 pandemic control measures were lifted, we observed a large increase in iGAS in children and adults. We wanted to understand whether this increase in 2022–early 2023 could be attributed to re-emerging viral infections like chickenpox and influenza, known to increase iGAS risk.

**What have we learnt from this study?**

We found that over 50% of iGAS skin and soft tissue infection and pneumonia/sepsis in children aged 0–5 years could be attributed to varicella and respiratory viruses including influenza over the study period between January 2010 and March 2023. However, these viral infections could only partly explain the high iGAS incidence after the pandemic. For adults, only 13% of iGAS pneumonia/sepsis could be explained by viral infections.

**What are the implications of your findings for public health?**

Public health interventions aimed to control chickenpox and influenza, such as vaccination, might reduce the burden of iGAS. However, such interventions would likely not suffice to reduce the iGAS incidence to pre-pandemic levels.

## Introduction

In 2022–23, an increase in the number of invasive group A streptococcal (iGAS) infections was reported by several countries, including the Netherlands [1,2]. While many infectious diseases exhibited re-emergence after the lifting of COVID-19 control measures, the re-emergence of iGAS seemed disproportionate to a mere catch-up effect from 2020 to 2021 [1]. An iGAS surge was initially observed among young children, showing a sevenfold increase in iGAS infections among children aged 0–5 years in the first half of 2022 compared with 2016–19 [1]. In particular, the high number of cases presenting with necrotising fasciitis was unprecedented. From November 2022 onward, a further increase in iGAS infections occurred, both in children and in adults, with pleural empyema and pneumonia observed more than expected, according to reports by clinicians. This pattern was also observed in the United Kingdom (UK), France, Spain, Denmark and Portugal [3-7].

Specific viral infections increase the risk of iGAS infection. For children, varicella (chickenpox) is a well-known risk factor for developing iGAS infection [8], as well as a more severe iGAS disease course [7,9]. Influenza infection is also a known risk factor for iGAS infection [10,11]. Influenza A and varicella, like iGAS, reached a high incidence in early 2022. A Dutch paediatric survey showed that varicella preceded or coincided with 33% of iGAS cases, and that 18% of iGAS infections were preceded by confirmed influenza infection [12]. Also, Nygaard et al. reported that in Denmark during the 2022–23 season, 22% of paediatric iGAS cases were preceded by varicella, and 47% by upper respiratory tract infection [7]. Surveillance data from England showed respiratory syncytial virus (RSV) and human metapneumovirus (hMPV) to be the most common co-infecting viruses in paediatric GAS in October–November 2022 [3]. In addition, a Portuguese study on paediatric iGAS between September 2022 and May 2023 reported that varicella and upper respiratory infection precede iGAS in 24.6% of cases [5]. As varicella is mostly associated with GAS skin and soft tissue infection (SSTI) and both varicella and respiratory viruses are risk factors for GAS pneumonia or sepsis [4,7,12], we hypothesised that increased viral infections could explain the observed high incidence of GAS SSTI (for varicella) and GAS pneumonia and sepsis (for varicella and respiratory viruses).

To assess the possible contribution of re-emergence of these viral infections on iGAS, we quantified the temporal association between varicella and GAS SSTI in children aged 0–5 years, and the association between varicella, influenza A, influenza B, RSV, hMPV and severe acute respiratory syndrome coronavirus 2 (SARS-CoV-2) and GAS pneumonia/sepsis in children aged 0–5 years old and in adults (excluding varicella) in the Netherlands. We also wanted to assess whether there were differences in the fraction of GAS SSTI and pneumonia/sepsis attributable to predisposing viral infections in the outbreak period 2022–23 compared with the pre-COVID-19 pandemic period 2010–19 and the pandemic years 2020–21, and whether the high incidences of predisposing viral infections provide a sufficient explanation for the high GAS SSTI and pneumonia/sepsis incidence observed in 2022–23.

## Methods

### Study design

We performed time-series regression analysis using separate existing registries of the different infectious diseases in the Netherlands. Our study period includes January 2010–March 2023. Our study quantifies temporal associations between numbers of GAS infections and numbers of viral infections 0–4 weeks prior. If a causal link between these types of infections is assumed, these associations can be interpreted as attributable proportions.

### Data sources

From the specified data sources for each infection, a dataset was generated with weekly values of determinants and outcomes, ranging from week 1 2010 (4 Jan 2010) to week 13 2023 (27 Mar 2023).

#### iGAS infections

We used the weekly number of GAS cultures from specific clinical specimens extracted from the database of the Dutch Infectious Diseases Surveillance Information System – Antimicrobial Resistance (ISIS-AR), as a measure for the number of iGAS infections [13]. Based on the year and month of birth, weekly numbers of isolates were obtained for children aged 0–5 years and for adults aged 18 and older. Ages 6 to 17 were not included in this study, because of low numbers and a presumed different dynamic of predisposing viral infections compared with young children and adults. 

The numbers of GAS pneumonia/sepsis were approximated by the number of GAS isolates cultured from blood or the lower airway. As a proxy for the number of GAS SSTIs, we extracted the number of GAS isolates cultured from skin, wounds or pus. As these are normally non-sterile sites, these cultures include both invasive and non-invasive GAS infections. In general, culture is not standard care for non-invasive infections in the Netherlands. Moreover, the majority of invasive SSTI are not confirmed by blood culture: only 37% of notified cases of GAS necrotising fasciitis during the study period were confirmed by blood culture. Therefore, these culture sites were chosen to capture the most severe SSTI, despite not being specific for iGAS.

Laboratories participating in the ISIS-AR network provide data for all isolates from medical routine diagnostics for which antibiotic susceptibility tests were performed, regardless of the pathogen. The database contains data from all types of healthcare facilities, including hospitals, primary care and long-term care facilities. In case of multiple isolates per patient within 3 months, we included only the first isolate in our analysis based on the arbitrary assumption that multiple isolates within 3 months stem from the same infection episode. The number of laboratories participating in ISIS-AR gradually increased during the study period, from an average of 32 laboratories per week in 2010 to 42 in 2014, after which the weekly number of participating laboratories stabilised. The number of laboratories contributing data per week is provided in Supplementary Figure S1. The total number of laboratories in the Netherlands decreased during the study period from ca 60 in 2010 to 50 in 2023. Because the catchment population of participating laboratories was unknown, a true population coverage could not be obtained. 

While iGAS is notifiable in the Netherlands, the notification criteria have changed over the study period and only include all iGAS since January 2023 [1]. Therefore, notifications do not provide a useful time series of iGAS.

#### Non-invasive GAS infections

The Nivel primary care database is a surveillance system monitoring general practitioner (GP) visits for a number of infectious disease syndromes [14]. From this database, the weekly number of visits by children aged 0–4 years with diagnosis streptococcal pharyngitis/scarlet fever (International Classification of Primary Care (ICPC) [15] code R72, henceforth ‘non-invasive GAS infection’) per 100,000 population was obtained as a proxy for the incidence of non-invasive GAS infections in young children. This time series was used as a representation of the GAS circulation in the general population.

#### Varicella zoster virus infections

The number of GP visits for varicella zoster (ICPC code A72) by children aged 0–4 years was obtained per week from the Nivel primary care database.

#### Respiratory viruses

The weekly numbers of detections of influenza A, influenza B, RSV and hMPV were obtained from the Dutch virological weekly report [16]. This is a surveillance system collecting aggregated numbers of virus detections per laboratory per week and does not contain any patient-level information.

#### SARS-CoV-2

Since the emergence of SARS-CoV-2, the testing policy has been highly variable, which would severely confound any time series based on laboratory detection. Therefore, the national average SARS-CoV-2 RNA load per 100,000 inhabitants from wastewater surveillance was used as a proxy for SARS-CoV-2 infections in the population [17]. The first SARS-CoV-2 infection in the Netherlands was detected on 27 February 2020. Data are available from 30 March 2020. The natural logarithm of the RNA load served as indicator of SARS-CoV-2 incidence. 

### Model assumptions and construction

The model was based on the following assumptions: (i) We assumed the iGAS counts in week *t* followed a negative binomial distribution, with expected value *µ_iGAS_*_,_ *_t_* and overdisperson parameter *ϕ*. The variance is given by *µ_iGAS_*_,_ *_t_* (1 + *µ_iGAS_*_,_ *_t_*/*ϕ*). The overdispersion parameter was treated as a nuisance parameter and assumed to be constant over the study period; (ii) We assumed changes in iGAS count to be attributable not only to circulation of non-invasive GAS and viruses, but also to unmeasured factors such as changes in population immunity and in the virulence of circulating GAS types. To allow for such explanatory unmeasured factors, a large-scale time trend was included. The large-scale trend term was modelled by penalised B-splines [18], reparametrised using an orthogonal decomposition to increase computational efficiency [19]. To ensure the trend was always positive, the exponent was taken; (iii) We assumed that antibiotic susceptibility was always tested for GAS isolates, as is the standard of care; (iv) We further assumed that all laboratories had roughly equal probabilities of contributing GAS isolates to the data. This weekly expected value of iGAS count was multiplied with the known proportion of laboratories reporting to ISIS-AR that week relative to the maximum number in the dataset (n = 45; see Supplementary Figure S1 for the number of laboratories per week), because the lower the number of participating laboratories, the less iGAS cases would be expected in the data. In other words, the weekly attributions were corrected for the time-varying completeness of the surveillance data; (v) The pathogen-specific regression parameters were assumed to be constant during the study period. Two exceptions are made. Firstly, because we worked with the natural logarithm of the RNA load as indicator of SARS-CoV-2 incidence, *β_SARS-CoV-2_* describes how much the expected iGAS count changes if the lag-weighted RNA load is a factor *e* larger. Secondly, the regression coefficient of influenza A, *β_infA_*, was allowed to vary between influenza seasons (piece-wise constant between July of one year to June next year), since it is known that the severity of influenza varies between seasons and in a previous study, we found that the association between influenza A and iGAS also varied between seasons [11]; (vi) We assumed the included weekly counts of the pathogens to be possibly associated with iGAS in the same week and the 4 following weeks. Supplementary Table S1 presents the weights per lag-week for all three models.

The weekly expected value *µ_iGAS_*_,_ *_t_* was written as the sum of a large-scale time trend *µ_trend_*_,_ *_t_* and pathogen specific contributions *µ_path_*_,_ *_i_*_,_ *_t_*, where *i* is the *i*-th pathogen. The weekly contribution of each pathogen to the expected iGAS counts is given by a linear function as the weighted sum of the reported pathogen incidence in current week *t* to 4 weeks back *t* − 4: *µ_path_*_,_ *_i_*_,_ *_t_* = Σ*_j_*  _=_ *w_i_*_,_ *_j_ β_path_*_,_ *_i_ path_i_*_,_ *_t_*_-_ *_j_*, *j* = 0,…, 4. The weights *w_i_*_,_ *_j_* were included to model possible lag-effects of pathogen *i* [20], and sum up to one. The weights are pathogen-specific and were assumed to be constant during the study period. Since we explicitly used the identity link function, the regression parameters *β_path_*_,_ *_i_* describe how much the expected iGAS count changes if the lag-weighted incidence of pathogen *i* changes one unit. To ensure attributions are always positive, *β_path_*_,_ *_i_* was forced to be greater than zero. We did not include any harmonic terms in the models. Instead, as a proxy for seasonality of GAS transmission, we included the number of consultations for non-invasive GAS syndromes.

All statistical analyses were carried out in R [21]. Because of the restrictions on the parameters (both the regression coefficients and the weights), the models were formulated in the Bayesian framework using priors. The models were fitted in Stan [22]. The regression parameters were given half-normal(0, 1) distributions, the weights were given a Dirichlet(0.2, 0.2, 0.2, 0.2, 0.2) distribution, and the overdispersion parameter *ϕ* was given an exponential(0.1) distribution.

## Results

**Figure 1:**
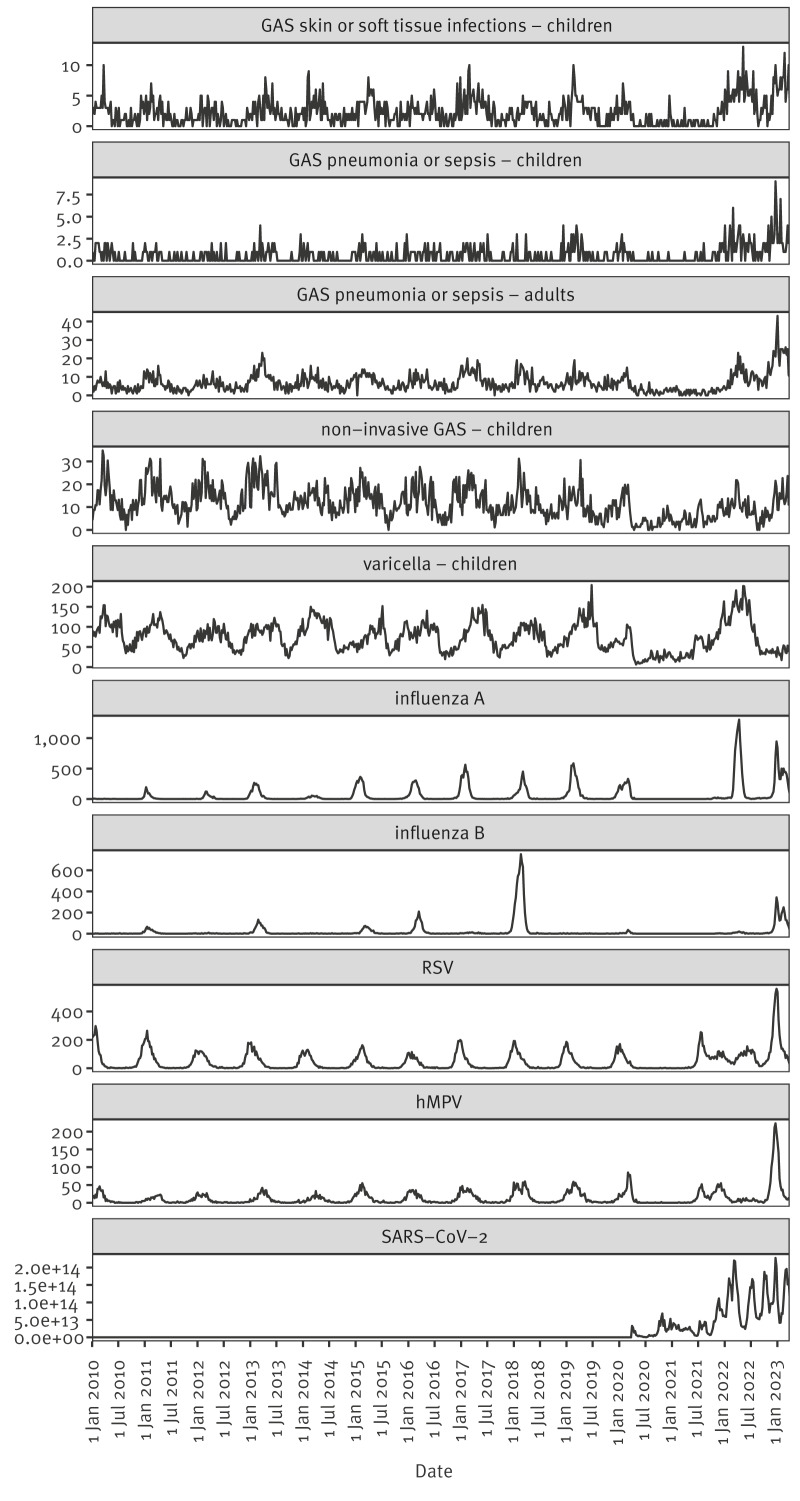
Observed weekly numbers of GAS skin and soft tissue infections in children, GAS pneumonia or sepsis in children and adults, varicella and non-invasive GAS infection consultations for children, and detections of respiratory viruses, the Netherlands, January 2010–March 2023

GAS: group A streptococcus; hMPV: human metapneumovirus; RSV: respiratory syncytial virus; SARS-CoV-2: severe acute respiratory syndrome coronavirus 2.

Over the entire study period, 1,595 GAS SSTI cases among children aged 0–5 years were included. For GAS pneumonia/sepsis, 446 cases among children aged 0–5 years, and 4,643 adult cases were included. The observed time series are shown in Figure 1. All GAS time series showed a winter seasonality, although this is less clear for pneumonia/sepsis in children given small numbers. The numbers of GAS SSTI and pneumonia/sepsis as well as GP consultations for non-invasive GAS syndromes were lower throughout 2020 and 2021. The numbers of GAS SSTI and pneumonia/sepsis in 2022 exceeded the peaks from seasons before 2020, in line with the trend seen in the national iGAS notifications [1]. However, non-invasive GAS GP consultations had returned to the typical levels observed before the pandemic. For most viruses, the typical seasonality was interrupted in 2020 and 2021 concurrent with COVID-19 public health and social control measures. Respiratory syncytial virus and hMPV started to re-emerge already in 2021, while influenza A re-emerged in 2022 and influenza B in 2023. Varicella GP consultations surged in the first months of 2022, followed by a very low incidence at the start of 2023 (Figure 1).

GAS skin and soft tissue infections (n = 1,606) and GAS pneumonia or sepsis cases in children (n = 449) aged 0–5 years; GAS pneumonia or sepsis cases in adults (n = 4,658) aged ≥ 18 years. For varicella (n = 51,596) and non-invasive GAS infection (n = 8,059), consultations were in children aged 0–4 years. The total number of detections for respiratory viruses (all ages) were as follows: influenza A (n = 42,966), influenza B (n = 13,128), RSV (n = 31,956), hMPV (n = 8,949). SARS-CoV-2 data are average sewage RNA load of severe acute respiratory syndrome coronavirus 2 per 100,000 inhabitants.

### GAS skin and soft tissue infections and varicella in children

**Figure 2:**
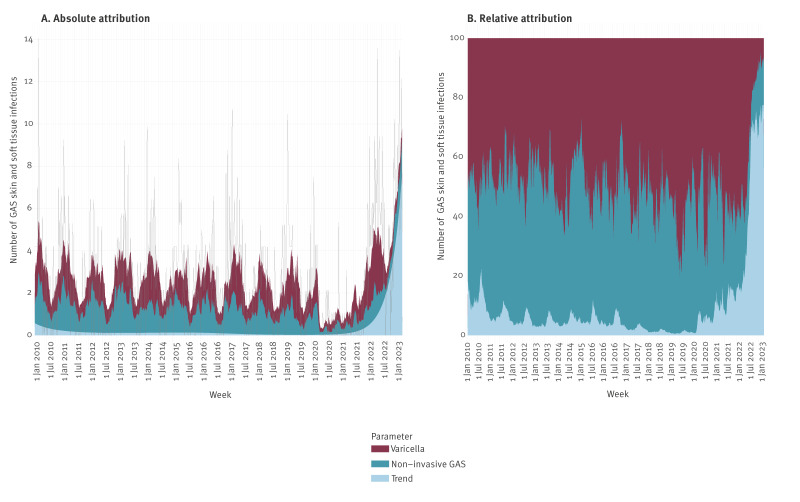
Estimated weekly absolute and relative attributions of GAS skin and soft tissue infections in children aged 0–5 years to varicella, non-invasive GAS and a long-term time trend, the Netherlands, January 2010–March 2023 (n = 1,595)

The estimated absolute number and proportion of GAS SSTI attributable to varicella and non-invasive GAS circulation are presented in Figure 2 and Table 1. From 2010 to 2019, the proportion of GAS SSTI estimated to be attributable to varicella fluctuated around 50%. Non-invasive GAS circulation was estimated to account for around 40% of the GAS SSTI incidence, with the remaining 10% not attributable to varicella nor non-invasive GAS; this remaining unexplained proportion of GAS SSTI is reflected in the large-scale time trend (‘trend’ parameter). In the first half of 2022, around 50% of GAS SSTI was still estimated to be attributable to varicella. From July 2022 to March 2023, the fraction attributable to varicella diminished rapidly, and the proportion of GAS SSTI not explained by varicella or non-invasive GAS (‘trend’ parameter) increased to 70%. Overall from January 2022 to March 2023, 34% (95% CI: 24–43) of GAS SSTI was attributable to varicella; a lower proportion than in 2010–19 and 2020–21.

GAS: group A streptococcus.

The grey line in panel A shows the observed number of GAS skin and soft tissue infections.

**Table 1:** GAS skin and soft tissue infections in children aged 0–5 years, attributed to varicella, the Netherlands, January 2010–March 2023 (n = 1,595)

Parameter	2010–19				2020–21				January 2022–March 2023				January 2010–March 2023 (overall)			
	Absolute		Relative		Absolute		Relative		Absolute		Relative		Absolute		Relative	
	n	95% CI	%	95% CI	n	95% CI	%	95% CI	n	95% CI	%	95% CI	n	95% CI	%	95% CI
Varicella	671	468–868	50	36–64	76	53–98	55	40–69	93	65–121	34	24–43	840	586–1,088	50	35–63

CI: confidence interval; GAS: group A streptococcus.

Absolute numbers are summarised per period, while the relative attributions are averaged over the period.

### GAS pneumonia/sepsis and predisposing virus infections in children

**Figure 3:**
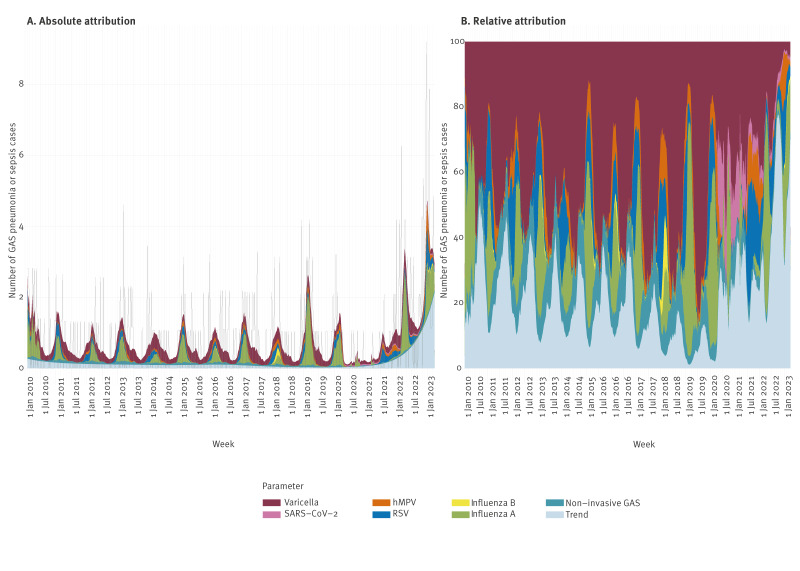
Estimated weekly absolute and relative attributions of GAS pneumonia or sepsis in children aged 0–5 years to varicella, SARS-CoV-2, hMPV, RSV, influenza B, influenza A, non-invasive GAS and a long-term time trend, the Netherlands, January 2010–March 2023 (n = 446)

GAS: group A streptococcal infections; hMPV: human metapneumovirus; RSV: respiratory syncytial virus; SARS-CoV-2: severe acute respiratory syndrome coronavirus 2.

The estimated absolute and relative number of GAS pneumonia/sepsis in children aged 0–5 years attributable to predisposing viruses are presented in Figure 3 and Table 2. In our model, varicella, influenza A, RSV and hMPV all provided relevant contributions to GAS pneumonia/sepsis cases in children aged 0–5 years, accounting for high attributable fractions during the seasonal peaks of these viruses (Figure 3B). The largest attributable fraction over the entire study period was estimated for varicella, with 43% (95% CI: 22–59), followed by influenza A (12%; 95% CI: 8–16). Over the period January 2022–March 2023, the average attributable proportions were 18% (95% CI: 9–27) for varicella, 17% (95% CI: 7–28) for influenza A, 10% (95% CI: 1–20) for RSV and 4% (95% CI: 0–11) for hMPV. SARS-CoV-2 did not seem to be a risk factor of concern for GAS pneumonia/sepsis incidence among children aged 0–5 years, with only an estimated five cases attributable despite very high rates of SARS-CoV-2 infections. Despite high incidences of respiratory viruses, we estimated the number of GAS pneumonia/sepsis not attributable to predisposing viruses or non-invasive GAS circulation to have increased to 46% (95% CI: 28–61) in January 2022–March 2023, compared with 18% (95% CI: 7–36) during 2020–19 (Figure 3A, ‘trend’ parameter).

The grey line in panel A shows the observed number of GAS pneumonia or sepsis cases.

**Table 2:** GAS pneumonia or sepsis in children 0–5 years old, attributed to predisposing viral infections, the Netherlands, January 2010–March 2023 (n = 446)

Parameter(s)	2010–19				2020–21				January 2022–March 2023				January 2010–March 2023 (overall)			
	Absolute		Relative		Absolute		Relative		Absolute		Relative		Absolute		Relative	
	n	95% CI	%	95% CI	n	95% CI	%	95% CI	n	95% CI	%	95% CI	n	95% CI	%	95% CI
Varicella	130	62–191	47	24–66	14	7–21	34	16–52	18	9–27	18	9–27	163	78–239	43	22–59
Influenza A	76	48–110	11	8–16	11	3–21	11	5–17	33	11–62	17	7–28	120	79–166	12	8–16
Influenza B	3	0–11	1	0–2	0	0–0	0	0–0	1	0–3	0	0–1	4	0–15	1	0–2
RSV	34	5–69	8	1–15	8	1–15	9	1–18	14	2–28	10	1–20	55	7–112	8	1–16
hMPV	19	1–50	4	0–12	5	0–13	6	0–15	7	0–19	4	0–11	31	1–82	5	0–12
SARS-CoV-2^a^	NA		NA		3	0–10	13	0–34	2	0–8	2	0–8	5	0–18	8	0–23
Respiratory viruses combined	132	97–172	24	18–31	26	15–40	38	23–57	57	32–87	34	20–49	216	158–277	27	20–35
Respiratory viruses + varicella combined	263	191–323	72	48–89	41	29–54	71	51–88	75	49–106	52	37–70	379	286–459	70	49–86

CI: confidence interval; GAS: group A streptococcal infections; hMPV: human metapneumovirus; NA: not applicable; RSV: respiratory syncytial virus; SARS-CoV-2: severe acute respiratory syndrome coronavirus 2.

^a^ The relative attributions to SARS-CoV-2 were averaged over April–December 2020 (for the year 2020) and over April 2020–March 2023 (overall).

Absolute numbers are summarised per period, while the relative attributions are averaged over periods.

### GAS pneumonia/sepsis and respiratory virus infections in adults

**Figure 4:**
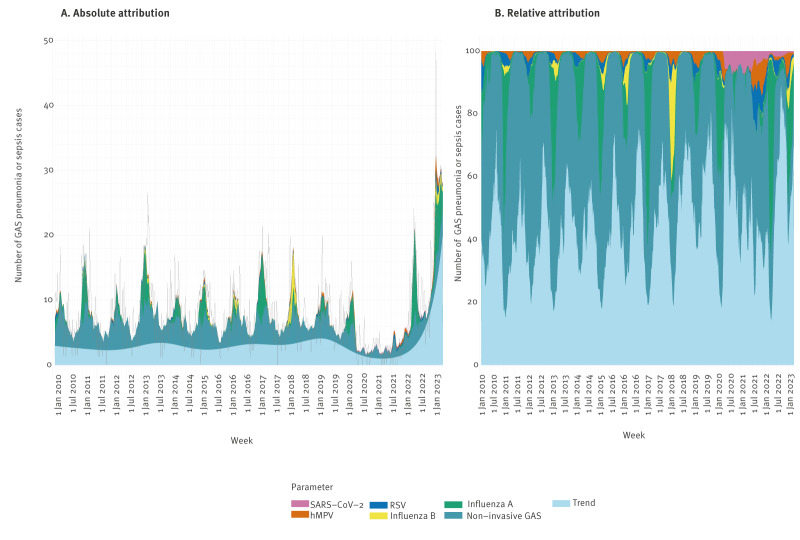
Estimated weekly absolute and relative attributions of GAS pneumonia or sepsis in adults to SARS-CoV-2, hMPV, RSV, influenza B, influenza A, non-invasive GAS and a long-term time trend, the Netherlands, January 2010–March 2023 (n = 4,643)

GAS: group A streptococcal infections; hMPV: human metapneumovirus; RSV: respiratory syncytial virus; SARS-CoV-2: severe acute respiratory syndrome coronavirus 2.

The estimated absolute number and proportion of GAS pneumonia/sepsis among adults attributed to respiratory viruses are presented in Figure 4 and Table 3. Overall, the proportion attributable to the combined respiratory viral infections examined is lower in adults (13%; 95% CI: 10–16) than in children aged 0–5 years (34%). Of the included respiratory viruses, influenza A accounted for the largest attributable fraction of GAS pneumonia/sepsis in adults with 7% (95% CI: 6–9) overall, varying between 5% in 2020–21 to 17% in January 2022–March 2023 (95% CI: 11–23). Similar to our findings in children, only a small fraction of GAS pneumonia/sepsis in adults could be explained by SARS-CoV-2 in January 2022–March 2023 (1%; 95% CI: 0–4). The fraction of GAS pneumonia/sepsis not attributable to respiratory viruses or non-invasive GAS was 45% (95% CI: 37–53) overall during the study period, increasing to 53% (95% CI: 45–60) in January 2022–March 2023.

The grey line in panel A shows the observed number of GAS pneumonia or sepsis cases.

**Table 3:** GAS pneumonia or sepsis in adults, attributed to respiratory virus infections, the Netherlands, January 2010–March 2023 (n = 4,643)

Parameter(s)	2010–19				2020–21				January 2022–March 2023				January 2010–March 2023 (overall)			
	Absolute		Relative		Absolute		Relative		Absolute		Relative		Absolute		Relative	
	n	95% CI	%	95% CI	n	95% CI	%	95% CI	n	95% CI	%	95% CI	n	95% CI	%	95% CI
Influenza A	416	292–552	7	5–9	42	15–72	5	3–8	210	110–317	17	11–23	668	489–851	7	6–9
Influenza B	83	21–152	1	0–2	1	0–2	0	0–0	25	6–45	1	0–3	109	27–199	1	0–2
RSV	61	3–177	1	0–3	14	1–39	3	0–8	25	1–71	3	0–8	99	4–287	2	0–5
hMPV	61	3–164	1	0–3	16	1–44	3	0–8	23	1–63	2	0–6	100	4–271	2	0–4
SARS-CoV-2^a^	NA		NA		12	0–38	5	0–16	9	0–29	1	0–4	20	0–66	3	0–11
Respiratory viruses combined	621	477–773	11	8–13	85	48–127	16	9–26	291	195–399	25	18–32	997	795–1,217	13	10–16

CI: confidence interval; GAS: group A streptococcal infections; hMPV: human metapneumovirus; RSV: respiratory syncytial virus; SARS-CoV-2: severe acute respiratory syndrome coronavirus 2.

^a^ The relative attributions to SARS-CoV-2 are averaged over April–December 2020 (for the year 2020) and over April 2020–March 2023 (overall).

Absolute numbers are summarised per period, while the relative attributions are averaged over periods.

## Discussion

Our study aimed to quantify the proportions of GAS SSTI in children and GAS pneumonia/sepsis in children and adults, attributable to the circulation of several common viral infections in the Netherlands from January 2010 through March 2023. Our model results provide a quantitative estimation of the proportion of iGAS cases that could be explained by re-emergence of predisposing viral infections during the period of excessive iGAS incidence in 2022–23. 

The attributable proportion of GAS SSTI and pneumonia/sepsis in children to varicella per year was around 50% before the pandemic. The high varicella incidence in 2022 after 2 years of very low incidence provided a likely explanation for the high iGAS incidence observed among children in the Netherlands in early 2022 [1]. However, the proportions of iGAS attributable to varicella were lower in 2022–March 2023. In early 2023, the varicella incidence was very low, but the number of GAS SSTI and pneumonia/sepsis remained elevated above pre-pandemic levels. Therefore, varicella does not provide a sufficient explanation for the increase in GAS SSTI observed among children in 2022–23.

The surge in GAS pneumonia/sepsis in children and adults was partly attributable to predisposing viral infections, in line with clinical observations of GAS pneumonia and pleural empyema with viral co-infections [12,23]. While the proportion attributable to respiratory virus infections was quite high in 2022–23 (34% for children, 25% for adults), a large fraction of GAS pneumonia/sepsis cases could not be attributed to either viruses or non-invasive GAS infections. SARS-CoV-2 does not seem to be an important risk factor for GAS pneumonia/sepsis in either children or adults; the relatively high attributable fraction of GAS pneumonia/sepsis in children averaged over the study period is mainly influenced by large fractions in 2020 and 2021, which are based on low case numbers.

Our results are in line with other European studies quantifying predisposing viral infection in iGAS cases during the 2022–23 surge. Nygaard et al. found 22% of Danish paediatric cases of soft tissue GAS infection had varicella zoster infection, but this proportion was not significantly higher compared with 2016–19 [7]. The same phenomenon was observed for upper respiratory tract infection (47% of paediatric iGAS cases in 2022–23). Despite these relatively constant proportions, the incidence of iGAS preceded by varicella had increased 4.6-fold in 2022–23 compared with 2016–19, and for upper respiratory tract infection this increase was 3.6-fold. This confirms our finding that surges of predisposing viruses can partly explain the iGAS increase. In Denmark, England and Scotland, paediatric iGAS cases with hMPV co-infections were noted in 2022–23, together with our results highlighting hMPV as a possibly underappreciated predisposing viral infection [3,7,24].

The large proportion of GAS SSTI and pneumonia/sepsis, both in children and adults, that remains unexplained by respiratory viruses or non-invasive GAS between January 2022 and March 2023, and attributed to the large-scale time trend parameter, suggests an unmeasured risk factor increasing in the end of the study period. This might relate to virulence changes in circulating GAS. In 2019, Lynskey et al. first described a new *emm*1 lineage among GAS, called M1_UK_, showing increased toxin production in vitro and replacing the previous *emm*1 strain (M1_global_) of GAS [25]. In 2019 in the Netherlands, 24% of *emm*-typed GAS isolates were *emm*1, of which 64% pertained M1_UK_ [26]. At the end of 2022, the proportion of *emm*1 among iGAS isolates increased rapidly from 32% in November to 68% in December [1] and remained above 50% up to the end of our study period. Rümke et al. showed that M1_UK_ had almost completely replaced M1_global_ strains in the Netherlands in 2022–23 [27]. Further evolution of M1_UK_, with acquisition of additional virulence genes, has been described [28], as well as another *emm*1.0 variant emerging in Denmark [29]. Increased virulence of GAS because of emerging variants may well have contributed to the increase in iGAS seen in 2022–23, thereby (partially) accounting for the unexplained proportions. Alternatively, increased susceptibility in the population from reduced exposure (‘immunity debt’) during the COVID-19 pandemic has been proposed as an explanation for the high iGAS incidence. However, such a generally increased susceptibility would likely also be reflected by high numbers of non-invasive GAS syndrome consultations, which we did not observe.

In the Netherlands, vaccination against varicella is not included in the national childhood immunisation programme. Seasonal influenza vaccination is offered to adults aged 60 years and older, and to persons of any age with certain medical conditions. Uptake of the seasonal influenza vaccine among eligible persons was estimated at 58% in 2021 [30]. Our results suggest that introducing varicella vaccination or expanding influenza vaccination eligibility could have an effect on iGAS incidence. Indeed, studies from Israel and Canada have shown a decrease in iGAS incidence after introduction of varicella vaccination [31,32].

A strength of our study is the inclusion of lags 0–4 weeks of all pathogens on the outcomes, by using a distributed lag model. Unlike fixed lag models, which can be biased if lag choice misaligns with the true data generating process, distributed lag models mitigate this by considering a range of lags. The distributed lag model provides a data-driven way to identify and capture appropriate delayed effects [20]. Another strength is the length of the study period. This allowed for robust estimation of pre-pandemic associations for comparison to the 2022–23 situation. While associations between iGAS and both influenza and varicella have been described before, our study further quantifies the associations between iGAS and RSV, hMPV, and SARS-CoV-2 and their relative importance.

Our study has several limitations. Firstly, the proxies used for GAS SSTI and pneumonia/sepsis are imperfect. GAS cultures from skin, pus and wound are not specific for invasive GAS and may include cultures from patients with non-invasive disease such as impetigo or abscesses. Conversely, for some patients with severe SSTI, GAS is confirmed by blood culture only and is therefore not included in the SSTI numbers. Likewise, the blood and lower airway cultures are not specific for GAS pneumonia/sepsis. Therefore, it is difficult to interpret our results in absolute numbers of iGAS infections or disease burden attributable to the pathogens included. Also, as the catchment populations of the participating laboratories are unknown and can vary over time, our adjustment for the number of laboratories contributing to the data likely does not fully capture variation in coverage. Secondly, a limitation of the virus detection data for influenza A and B, RSV and hMPV is that these cannot be stratified into age groups and detections may be influenced by changes in testing policies, especially during the pandemic, affecting the time series. A third limitation of our study is the implicit assumption that the models were correctly specified. This assumption could not be fully tested. Results could change if we would have included other pathogens or excluded some of the pathogens for which we have calculated the attribution. This means that the model estimates should be interpreted with care. Also, in time series modelling, harmonic terms are often included to allow for seasonal baseline fluctuations in the outcome variable. As we included data on consultations for non-invasive GAS syndromes, which incorporates the winter seasonality of GAS infections in general, we chose not to include harmonic terms. Had we done so, possibly the proportions attributed to e.g. varicella and influenza would have been lower. On the other hand, the choice to model the long-term time trend using a penalised spline allowed for the absorption of unaccounted effects, thereby mitigating model misspecification. The flexibility of the spline term enables it to capture variations in the data that might not be adequately addressed by the predefined model structure. This absorption of unexplained variability into the spline term enhances the model's ability to fit the data accurately and reduces the potential for misrepresenting relationships. Had we used a less flexible time trend, such as a linear term or constant intercept, likely the attribution to mainly influenza A would have been larger, as the coefficient for this pathogen was allowed to vary per season. Finally, the fact that this is an ecological study of temporal associations is also a limitation. The associations we reported do not necessarily reflect causal effects, although such causality has been established by other studies. For example, effects of influenza A infection on iGAS infection and severity has been shown in murine studies [10,33]. 

## Conclusions

Our results point to relevant attributable fractions of GAS SSTI among children to varicella and of GAS pneumonia/sepsis to influenza A in adults and in children aged 0–5 years, and additional relevant attributable fractions of GAS pneumonia/sepsis in children to RSV and hMPV. However, the re-emergence of these viruses does not provide a sufficient explanation for the surge in iGAS infections in 2022–23 in either children or adults in the Netherlands. Other factors increasing iGAS risk, such as microbial virulence factor acquisition, may be at play.

## Supplementary Data

212000 Supplement
