# Supplementary material for: Attribution of invasive group A streptococcal infections (iGAS) to predisposing viral infections, the Netherlands, 2010 to 2023
Source: Euro Surveill. 2024 Oct 3;29(40):2300739. doi: 10.2807/1560-7917.ES.2024.29.40.2300739 (PMC11451131; doi:10.2807/1560-7917.ES.2024.29.40.2300739)
Supplement: Supplement [file 23-00739_GIER_Supplement.pdf]

This supplementary material is hosted by Eurosurveillance as supporting information alongside the article "Attribution of invasive group A streptococcal infections to predisposing viral infections, the Netherlands, 2010-2023", on behalf of the authors, who remain responsible for the accuracy and appropriateness of the content. The same standards for ethics, copyright, attributions and permissions as for the article apply. Supplements are not edited by Eurosurveillance and the journal is not responsible for the maintenance of any links or email addresses provided therein.

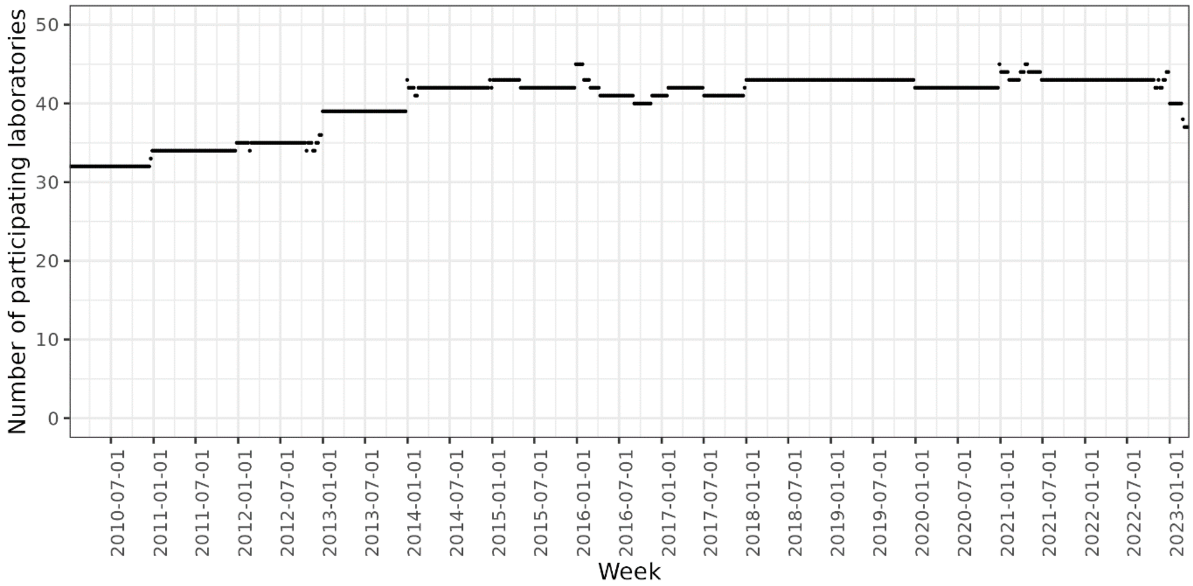

Figure S1. Number of laboratories contributing data to ISIS-AR per week during the study period (January 2010- March 2023).

Table S1. Lag weights (95% CI) for each of the pathogens. GAS; group A streptococcus; ni-GAS: non-invasive GAS; RSV: respiratory syncytial virus; hMPV; human metapneumovirus; SARS-CoV-2: severe acute respiratory syndrome coronavirus 2; SSTI: skin and soft tissue infections.

Model	Pathogen	Lag (weeks)				
		0	1	2	3	4
GAS pneumonia/sepsis - adults	ni-GAS	0.03 (0-0.19)	0.09 (0-0.31)	0.22 (0-0.45)	0.24 (0-0.47)	0.42 (0.21-0.63)
	Influenza A	0.57 (0.03-0.88)	0.13 (0-0.73)	0.04 (0-0.28)	0.04 (0-0.27)	0.22 (0-0.47)
	Influenza B	0.38 (0-0.98)	0.27 (0-0.97)	0.13 (0-0.84)	0.12 (0-0.77)	0.11 (0-0.66)
	RSV	0.14 (0-0.84)	0.15 (0-0.88)	0.22 (0-0.95)	0.26 (0-0.96)	0.23 (0-0.96)
	hMPV	0.28 (0-0.97)	0.17 (0-0.92)	0.19 (0-0.91)	0.19 (0-0.93)	0.16 (0-0.9)
	SARS-CoV-2	0.19 (0-0.92)	0.2 (0-0.94)	0.2 (0-0.94)	0.21 (0-0.95)	0.2 (0-0.95)
GAS pneumonia/sepsis – children 0-5	ni-GAS	0.15 (0-0.86)	0.25 (0-0.96)	0.22 (0-0.94)	0.16 (0-0.91)	0.22 (0-0.94)
	Influenza A	0.53 (0-0.96)	0.12 (0-0.75)	0.06 (0-0.45)	0.12 (0-0.62)	0.16 (0-0.59)
	Influenza B	0.2 (0-0.94)	0.18 (0-0.92)	0.19 (0-0.94)	0.21 (0-0.95)	0.23 (0-0.96)
	RSV	0.42 (0-0.99)	0.21 (0-0.96)	0.19 (0-0.95)	0.1 (0-0.73)	0.08 (0-0.56)
	hMPV	0.26 (0-0.97)	0.19 (0-0.93)	0.23 (0-0.96)	0.18 (0-0.94)	0.13 (0-0.84)
	SARS-CoV-2	0.2 (0-0.94)	0.2 (0-0.94)	0.2 (0-0.95)	0.2 (0-0.94)	0.2 (0-0.95)
	varicella	0.18 (0-0.9)	0.52 (0-0.99)	0.15 (0-0.84)	0.1 (0-0.71)	0.05 (0-0.39)
GAS SSTI – children 0-5	ni-GAS	0.38 (0-0.76)	0.25 (0-0.7)	0.22 (0-0.62)	0.07 (0-0.34)	0.08 (0-0.36)
	varicella	0.83 (0.43-1)	0.06 (0-0.38)	0.06 (0-0.34)	0.04 (0-0.25)	0.02 (0-0.16)
